# Risk Factors of Walking While Talking Decline in Older Adults: Central Control of Mobility and Aging Study

**DOI:** 10.1093/geroni/igab046.3188

**Published:** 2021-12-17

**Authors:** Oshadi Jayakody, Helena Blumen, Emmeline Ayers, Joe Verghese

**Affiliations:** 1 Albert Einstein College of Medicine, The Bronx, New York, United States; 2 Albert Einstein College of Medicine, New York, New York, United States

## Abstract

Background: Slow gait speed during walking while talking (WWT-speed) is associated with an increased risk of falls and dementia. Age-related changes in WWT-speed and associated risk factors, however, are poorly understood. This study examined 1) change in WWT-speed over time 2) factors associated with change in WWT-speed. Methods: A total of 431 older participants (M Age=76.8±6.4 years; M follow-up 4.5±2.3 years) enrolled in the Central Control of Mobility in Aging study were examined. WWT-speed was measured with a computerized walkway while participants recited alternate letters of the alphabet while walking. The following baseline measures were examined as risk factors: demographic [age, sex, education], medical [hypertension, diabetes, cardiac arrhythmias, history of stroke, Parkinson’s disease, kidney disease, arthritis, depression], cognitive [global cognition, executive function, processing speed], sensorimotor [balance, grip strength, vision], falls and frailty. Linear mixed effect models were used to examine 1) change in WWT-speed over time 2) risk factors of WWT-speed change. Results: WWT-speed declined over time (b -1.06, 95%CI -1.45, -.68) independent of baseline age, sex and education. Rate of WWT-decline was modified by age (b -.10, 95%CI -.17, -.03) and poorer balance (b -1.12, 95%CI -1.95, -.28). Lower scores in tests of global cognition and processing speed and, kidney disease predicted slow WWT-speed on average. Conclusion: Greater age and poorer balance accelerate WWT-speed decline while poorer global cognition, slow processing speed and kidney disease predicts slow WWT-speed. These factors may provide potential targets for future interventions to prevent decline in WWT-speed and associated adverse health outcomes.

